# Clinical correlates of symptom severity in skin picking disorder

**DOI:** 10.1016/j.comppsych.2017.07.001

**Published:** 2017-10

**Authors:** Jon E. Grant, Samuel R. Chamberlain

**Affiliations:** a Department of Psychiatry & Behavioral Neuroscience, University of Chicago, Chicago, IL, USA; b Department of Psychiatry, University of Cambridge, Cambridge and Peterborough NHS Foundation Trust (CPFT), UK

## Abstract

**Background:**

Skin picking disorder (SPD) remains poorly understood with limited data regarding its underlying pathophysiology and appropriate treatment choices. One approach to refining our treatment of SPD might be to better understand the range of illness severity and the clinical associations with severity.

**Methods:**

125 adults aged 18 to 65 with a primary, current DSM-5 diagnosis of SPD were assessed for the severity of their picking, using the Skin Picking Symptom Assessment Scale, and related mental health symptoms. To identify clinical and demographic measures associated with variation in disease severity, we utilized the statistical technique of partial least squares (PLS).

**Results:**

Greater SPD symptom severity was associated with higher Barratt attentional impulsiveness and motor impulsivity, higher Eysenck impulsivity, higher state anxiety/depression, having a current anxiety disorder, and having a lifetime substance use disorder.

**Conclusions:**

The present analysis is, to our knowledge, the most complete assessment of clinical variables and their relationship to illness severity in a sample of adults with SPD. Aspects of impulsivity and anxiety are both strongly associated with worse illness severity, and functional disability, in SPD. Treatment approaches should incorporate these as possible treatment targets when developing new treatment approaches to this disorder.

## 1 Introduction

Skin-picking disorder (SPD) is a disabling, under-recognized condition in which individuals repeatedly pick at their skin, leading to noticeable tissue damage. Newly formalized in the 5th edition of the Diagnostic and Statistical Manual of Mental Disorders (DSM-5), SPD has the following diagnostic criteria: recurrent picking resulting in skin lesions; repeated attempts to stop picking; and clinically significant distress or psychosocial impairment due to the picking [1]. Psychosocial impediment, reduced quality of life, and medical problems such as infections are common among individuals with SPD [2], [3].

Skin picking disorder remains poorly understood with limited data regarding its underlying pathophysiology and appropriate treatment choices [4]. There have been only five placebo-controlled trials of pharmacotherapy published for the treatment of SPD, and some have produced mixed results. Specifically, three of these studies examined selective serotonin reuptake inhibitors (SSRIs), with one (citalopram) exhibiting no difference from placebo, one (fluoxetine) demonstrating superiority to placebo, and one (fluoxetine) differentiating itself from placebo on a single outcome measure [5], [6], [7]. A single study reported significant benefits from the glutamate modulator *n*-acetyl cysteine versus placebo [8], whereas another study found that lamotrigine did not differentiate from placebo overall (though it possibly helped in a subset of patients with impaired cognitive flexibility) [9].

In terms of psychotherapy studies, there have been three randomized studies, one of a brief cognitive–behavioral therapy (CBT) intervention [10], one comparing habit reversal to wait-list [11], and a third study comparing self-help versions of habit reversal training (HRT) decoupling [12]. Although CBT and HRT appear promising, data are still lacking regarding optimal treatment duration and who with SPD would benefit most from which psychotherapy.

One approach to refining our treatment of SPD might be to better understand the range of illness severity and the clinical associations with severity. The DSM-5 criteria stipulate the minimal level of severity to meet diagnostic threshold (i.e. repeated behavior with resulting skin lesions) but do not provide details about severity levels and whether those can be meaningful in terms of treatment approaches. Thus the goal of this study was to identify clinical and demographic measures associated with variation in disease severity in a large sample of adults with SPD. Given the associations between impulsivity and skin picking [4], we hypothesized that greater skin picking symptom severity would be associated with a greater degree of impulsivity.

## 2 Methods

### 2.1 Subjects

Men and women aged 18 to 65 with a primary, current DSM-5 diagnosis of SPD were recruited by newspaper advertisements and referrals for neuroimaging or treatment studies. Exclusion criteria included: 1) unstable medical illness; 2) history of seizures; 3) lifetime history of bipolar disorder, dementia, or psychotic disorder; 4) current (past 3 months) substance use disorder; 5) current risk of suicide (defined as endorsing any symptom on the Columbia Suicide Severity Scale) [13]; and 6) current pregnancy or inadequate contraception in women of childbearing potential.

Data were collected from September 2011 to June 2012 at the University of Minnesota and then from December 2012 to the present time at the University of Chicago. The Institutional Review Boards for the University of Minnesota and the University of Chicago approved the studies and the informed consent procedures. After complete description of the studies and an opportunity to ask questions, participants provided written informed consent. This research was carried out in accordance with the principles of the Declaration of Helsinki. IND 108195 was assigned by the FDA.

### 2.2 Assessments

Demographics and clinical features of SPD were assessed with a semi-structured interview. The semi-structured interview included proposed diagnostic criteria for SPD as well as questions regarding the phenomenology of picking. After the publication of the DSM-5, all subjects were retrospectively assessed based on case notes, and all clearly met full diagnostic criteria. Race/ethnicity was defined by the study subjects and was included to learn more about this variable in SPD. Psychiatric comorbidity was assessed using the Structured Clinical Interview for DSM-IV (SCID) [14].

All participants were assessed for the severity of their picking and related mental health symptoms. The severity of SPD was assessed using the self-report Skin Picking Symptom Assessment Scale (SP-SAS) [15]. The SP-SAS is a self-report scale that has satisfactory test-retest reliability and satisfactory change over time [15]. The SP-SAS is scored from 0 to 24, with higher scores indicative of greater symptom severity.

Psychosocial functioning and depressive and anxiety symptoms were further assessed using the following valid and reliable measures: the patient-administered Sheehan Disability Scale (SDS) [16] and the clinician-administered Hamilton Anxiety Rating Scale (HAM-A) [17], and the clinician-administered Hamilton Depression Rating Scale (HAM-D) [18].

In addition, to examine impulsivity, each participant completed the Barratt Impulsiveness Scale 11 (BIS) [19] and the Eysenck Impulsiveness Questionnaire (EIQ) [20]. The BIS is a 30 question self-report measure that is designed to assess various aspects of impulsivity, yielding three total scores: attentional impulsivity, motor impulsivity, and non-planning impulsivity. The EIQ is a 54 question self-report measure comprised of three subscales: impulsivity, venturesomeness, and empathy.

### 2.3 Cognitive assessments

Cognitive assessments consisted of two previously validated tests taken from CANTABeclipse software. The choice of cognitive challenges was based on the clinical features of SPD. Previous research has found that individuals with SPD often exhibit significant deficits of motor inhibition and cognitive flexibility compared to healthy controls [21]. All testing was conducted in the same controlled environment to minimize confounding variables across subjects. The order of the tasks was fixed.

Cognitive flexibility, i.e., set-shifting, was measured using the using the Intra-dimensional/Extra-dimensional Shift Task (IED task) [22]. On the task, subjects were presented with two stimuli on-screen for each trial, and attempted to learn an underlying ‘rule’ about which stimulus was correct. After selecting a stimulus, the computer provided feedback as to whether the choice was right or wrong. Through this feedback, participants attempted to learn underlying rules. Once they had identified the underlying rule, the task changed the rule, in order to measure the ability of the person to exhibit flexible responding. Key outcome measure was the total number of errors made on the extra-dimensional shift stage (the key stage when high level cognitive flexibility is measured).

The stop-signal task (SST) was used to assess motor inhibition [23]. On this task, participants were instructed to respond to left- or right-facing arrows which appeared on a computer screen, one per time, in a rapid fashion. When an auditory ‘stop-signal’ (beep) occurred, participants attempted to suppress their motor response for that given trial. By varying the time between the arrow presentation and the stop-signal, the task estimated the time taken by the individual to suppress a pre-potent response, referred to as the stop-signal reaction time. Median response times for go trials were also recorded, as a measure of general psychomotor speed.

### 2.4 Data analysis

To identify clinical and demographic measures associated with variation in disease severity, we utilized the statistical technique of partial least squares (PLS) [24], [25], [26], [27]. PLS is a multivariate, iterative technique that constructs one or more latent factors (referred to as PLS components) that optimally explain variation in X and Y. The Y variable was total score on the SP-SAS and X variables were as follows: age, educational level, gender, age at first diagnosis, past treatment for SPD, history of grooming disorder or substance use disorder in one or more first-degree relatives, current smoking status (smoker or non-smoker), Hamilton Anxiety and Depression scale total scores, picking from multiple sites, presence of major depressive disorder, presence of any anxiety disorder, presence of any substance use disorder, presence of body dysmorphic disorder, presence of OCD, presence of ADHD, Barratt impulsiveness subscale scores (attentional, non-planning, and motor), Eysenck scores (impulsivity, empathy, venturesomeness), extra-dimensional set-shifting errors, and stop-signal reaction times (SSRTs). Unlike traditional regression, PLS is ideal in situations in which variables are correlated with each other; and when the number of variables is large in comparison to the number of cases, as was the case here [24], [25], [26], [27]. Analysis was conducted using JMP Pro software Version 13.0. Any missing data points were imputed automatically by JMP using study means. The PLS model was fitted using leave-one-out cross-validation (non-linear iterative partial least squares, NIPALS algorithm), and the optimal number of latent factors was selected by minimizing the predictive residual sum of the squares (PRESS). X variables significantly contributing to the model (i.e. explaining significant variance in disease severity) were identified on the basis of 95% confidence intervals for bootstrap distribution of the standardized model coefficients not crossing zero (*N* = 1000 bootstraps). To confirm or refute the clinical relevance of the PLS model, we examined whether latent factor score(s) on the model correlated significantly with Sheehan Disability scores across all participants.

## 3 Results

A total sample of 125 participants (mean age = 34.1 ± standard deviation 11.9 years; 87.2% female) were recruited. The participants reported a mean age at the onset of SPD of 12.9 (9.0) years. Most (92 [73.6%]) participants picked skin from multiple sites. The mean score on the SP-SAS was 28.7 (6.7) [range 13 to × 36] and the mean SDS score was 11.4 (6.7) [range 0 to 28]. Depression and anxiety symptoms were fairly low with mean scores on the (HAM-A) and (HAM-D) 4.4 (3.7) [range 0 to 20] and 4.6 (3.9) [range 0 to 21], respectively.

**Fig. 1:**
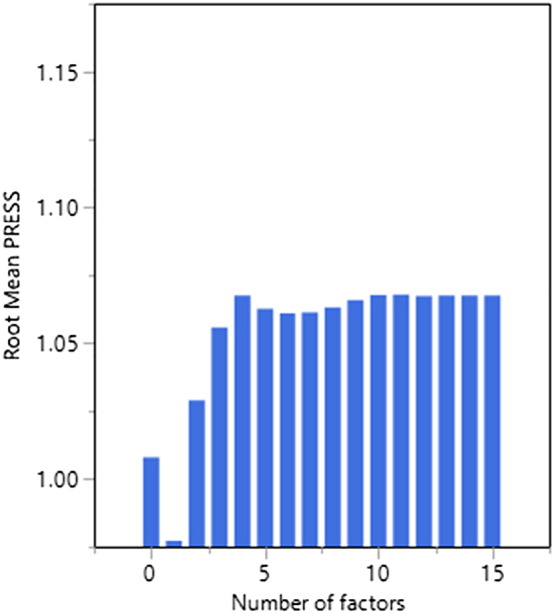
Predictive residual sum of the squares (PRESS) as a function of the number of latent factors.

Cross-validation showed that the optimal fit PLS model to minimize PRESS had one latent variable (Fig. 1) and hence this model was selected. This model accounted for total 9.6% of the variation in the clinical/demographic measures, and 25.5% of variation in disease severity (SP-SAS total scores).

**Fig. 2:**
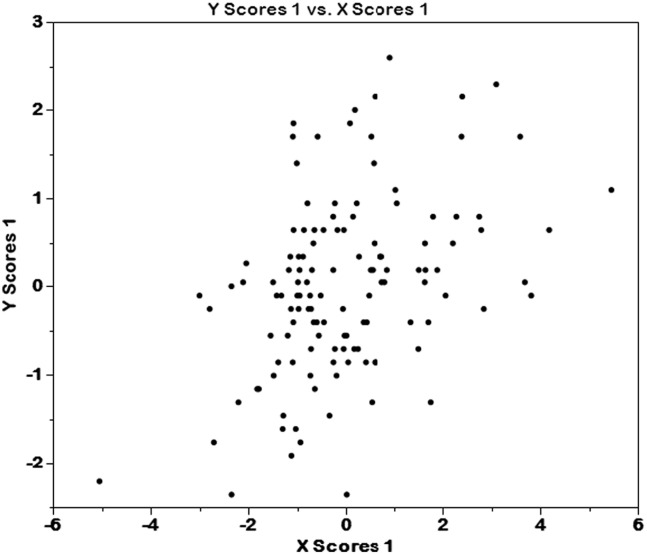
Distribution of PLS X and Y scores across individuals in the study.

The standardized model coefficients for each variable of interest are presented in Table 1. Variables with positive coefficients had a positive relationship with SP-SAS total scores, and vice versa. Those measures shown in bold and with an asterisk retained statistical significance by bootstrap, i.e. the 95% confidence interval of the bootstrap distribution of the model coefficient did not cross zero (Fig. 2). Thus, higher SPD symptom severity was associated with higher Barratt attentional and motor impulsivity, higher Eysenck impulsivity, higher state anxiety/depression, having a current anxiety disorder, and having a lifetime history of substance use disorder. Both X and Y PLS scores correlated significantly and positively with functional impairment on the Sheehan Disability Scale across all participants (Spearman's *r* = 0.2, *p* = 0.03; and *r* = 0.50, *p* < 0.001 respectively).

**Table 1:**
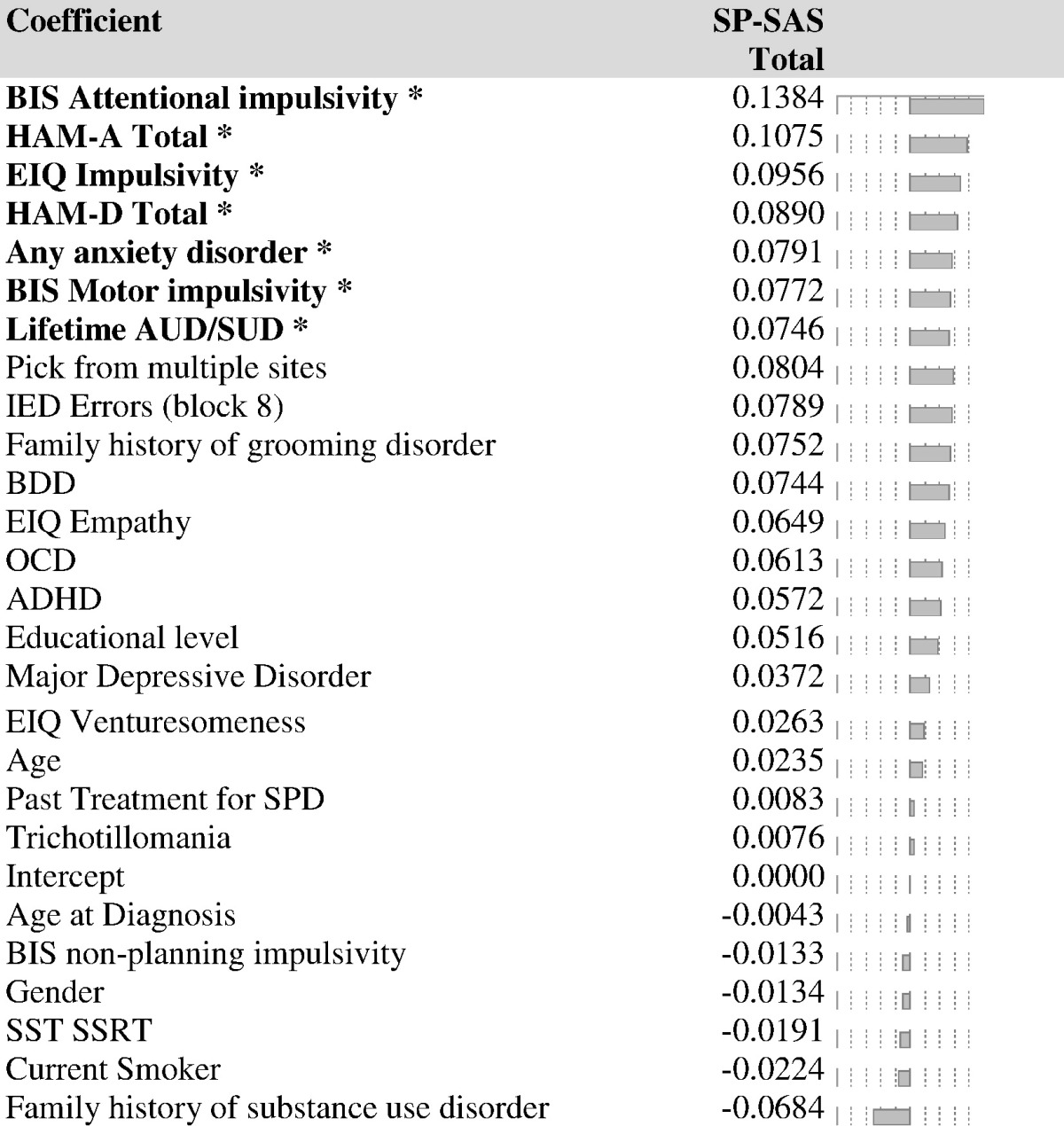
Standardized model coefficients for each X variable of interest in the optimal PLS model (one latent variable). *: statistically significant predictive variable by bootstrap.

## 4 Discussion

To our knowledge, this is the first study to examine the clinical correlates of illness severity in a large sample of adults with SPD. There were several important findings from this analysis, which was conducted using the statistical methodology of partial least squares (PLS), which best explained the co-variation between demographic/clinical measures and symptom severity by constructing a single latent factor from the data. Aspects of impulsivity (specifically domains of the BIS and the EIQ) were significantly and positively associated with illness severity, as were co-occurring history of substance addiction and elevated state symptoms of anxiety and depression. Crucially, we demonstrated that higher scores on the latent PLS component were significantly correlated with greater impairment on the Sheehan Disability Scale, confirming that the model was clinically relevant. One important point is that only certain aspects of impulsivity were positively correlated, not all types of impulsivity (for example, certain domains of the BIS and EIQ, as well as categorical ADHD and the cognitive measure of the SST were not significant in the PLS model). Secondly, no measure of compulsivity was significantly associated with illness severity in the model (that is, the IED block 8 errors and categorical OCD). Finally, although depressive symptoms were correlated with severity, rates of categorical major depressive disorder were not, though this may reflect the relative scarcity of clinical depression in the cohort.

Taken together, these data suggest that very specific clinical variables are associated with illness severity in SPD, and as such, may represent useful and specific targets for treatment interventions. The domains of attentional and motor impulsivity on the BIS were robustly associated with illness severity. Attentional impulsivity is characterized by the ability to sustain attention on a given task (for example, “I don't ‘pay attention’”), whereas motor impulsivity is related to the ability to control behavioral (for example, “I act on ‘impulse’”). The association with substance use disorders may also be related to these impulsivity constructs. These may be appropriate targets for cognitive behavioral therapy or even cognitive rehabilitation using specific computer tasks focusing on these particular cognitive domains of interest. Similarly, the centrality of impulsive measures in explaining variation in symptom severity in SPD suggests that medications capable of treating impulsivity would be worth exploring in clinical trials. For example, there have been no blinded trials of stimulant medication in SPD to date. Although the treatment of the picking alone may be sufficient to address some comorbid findings, the question arises as to whether simultaneously treating co-occurring substance use, for example, may be useful in decreasing the symptoms of SPD.

One surprising finding was that the BIS measurement of motor impulsivity showed significant associations with symptom severity while the SST did not. This seems contradictory to a previous report that SST was significantly different in adults with SPD compared to controls [28]. This discrepancy may be explained by the fact that the SST represents a trait marker that characterizes SPD but that it has no relationship to illness severity, whereas the BIS does. Alternatively, this discrepancy between the two measures may indicate that the concept of “motor impulsivity” is multidimensional and requires a more detailed understanding of motor impulsivity and its neurobiological underpinnings than relying on one laboratory-based measure that may be reductionist.

The present findings emphasize the importance of refining our subtyping of impulsivity. If researchers are able to specify the specific nature of impulsivity within people with SPD, clinicians may be able to design treatment plans that are specifically adapted to manage problems resulting from that particular form of impulsivity.

Anxiety symptoms and anxiety disorders were both positively associated with illness severity in this study. This seems consistent with previous research that has found high rates of anxiety disorders among those with SPD and has found that picking is often triggered by and in turn alleviates feelings of anxiety [29], [30], [31]. These findings provide further evidence that anxiety and picking may reflect a complex cycle of behavior and that approaches to anxiety alone may not be enough to eliminate picking but that treatment which ignores anxiety may be less effective.

Although this study represents a potentially beneficial approach to understanding SPD, there exist several limitations. Our approach of defining the statistical significance of individual measures in the PLS model by using bootstrap is quite conservative and so some variables may have been overlooked (false negatives). However, this approach does mean that one can have a high degree of statistical confidence in the significant results (low risk of false positive error). As with any such study, the current data cannot show that the findings would generalize to SPD patients presenting in other settings such as to family doctors. The proportion of variance accounted for was relatively modest and other unmeasured variables are likely to be important in being associated with disease severity. Genetic polymorphisms, information on upbringing/childhood trauma, as well as a broader range of biological measures (e.g. neuroimaging parameters) may all be valuable for future studies to consider.

The present analysis is, to our knowledge, the most complete assessment of clinical variables and their relationship to illness severity in a sample of adults with SPD.

From these findings, it seems that aspects of impulsivity and anxiety are both strongly associated with worse illness severity, and functional disability, in SPD. As such, treatment approaches should incorporate these as possible treatment targets when developing new treatment approaches to this disorder.
